# S100A8/A9 is the first predictive marker for neonatal sepsis

**DOI:** 10.1002/ctm2.338

**Published:** 2021-04-05

**Authors:** Sabine Pirr, Louise Dauter, Thomas Vogl, Thomas Ulas, Bettina Bohnhorst, Johannes Roth, Dorothee Viemann

**Affiliations:** ^1^ Department of Pediatric Pneumology Allergology, and Neonatology, Hannover Medical School Hannover Germany; ^2^ Institute of Immunology University of Münster Münster Germany; ^3^ Genomics and Immunoregulation LIMES‐Institute University of Bonn Bonn Germany; ^4^ Cluster of Excellence RESIST (EXC 2155) Hannover Medical School Hannover Germany

Dear Editor,

Neonatal sepsis is a leading cause of childhood mortality worldwide particularly affecting preterm infants, who are often exposed to empirical antibiotics since sepsis biomarkers lack sensitivity in this patient group and markers predicting the risk of sepsis have not been identified yet.^1^, ^2^ In our study, serum S100A8/A9 proved as an independent predictive marker of late‐onset neonatal sepsis (LOS) in preterm infants, which for the first time offers the opportunity to change current treatment policies by improving antibiotic stewardship and timely individualized therapeutic intervention.

Serum S100A8/A9 (also known as serum calprotectin) is an alarmin respective damage‐associated molecular pattern (DAMP) that is rapidly released from myeloid cells upon stress or cell damage, acting then as secondary amplifier of inflammation.^3^ In adults, the S100A8/A9 serum level is used as one of the most sensitive biomarkers in inflammatory processes.^4^ Its value as sepsis marker in neonates remains unclear and needs to be tested. On the other hand, we previously identified S100A8/A9 as an essential sepsis‐protective regulator of neonatal immunity^5^, ^6^ with serum levels being physiologically increased after birth.^7^ Thereby, significantly higher levels in term compared to preterm infants^6^ suggest that low S100A8/A9 in neonates is associated with an increased risk of sepsis. However, other parameters potentially influencing neonatal S100A8/A9 serum levels are ill defined, wherefore the value of S100A8/A9 as an independent predictive marker of neonatal sepsis also remains to be demonstrated.

A total of 289 preterm infants born below 32 gestational weeks were prospectively enrolled. In a case–control study, of 41 infants serum levels of S100A8/A9, C‐reactive protein (CRP) and interleukin 6 (IL‐6) were determined at the onset of neonatal sepsis and compared with 50 matched healthy controls (Table S1 and Methods in the Supporting Information). However, S100A8/A9 did not distinguish septic infants from healthy controls due to already elevated S100A8/A9 levels in nondiseased infants (Figure 1A). Consequently, serum S100A8/A9 did not outcompete sepsis marker like CRP and IL‐6 (Figure 1B–D) currently used in the clinical routine.

**FIGURE 1:**
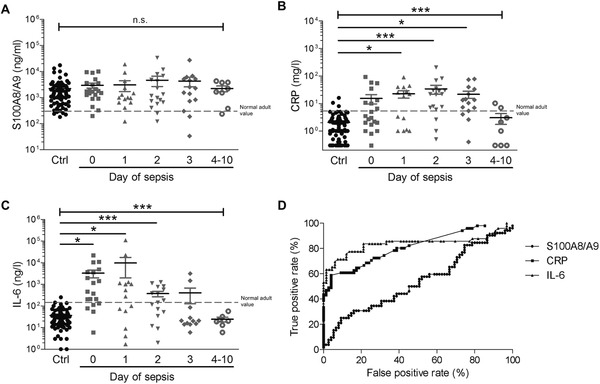
Kinetics of serum levels of S100A8/A9, CRP, and IL‐6 during neonatal sepsis in preterm infants. Serum levels of (A) S100A8/A9, (B) C‐reactive protein (CRP), and (C) interleukin 6 (IL‐6) were measured in preterm neonates during the course of neonatal sepsis (*n* = 41) with day 0 representing the day of first clinical suspicion of sepsis before start of antibiotic treatment and preterm infants matched for gestational and postnatal age, mode of delivery (MOD), sex, and birth weight (BW) (controls, Ctrl; *n* = 50). S100A8/A9 serum levels only slightly but not significantly increased at initial presentation of sepsis compared to control children due to the already high normal levels of S100A8/A9 in preterm control infants during the first 2 weeks of life (A). Levels did not change significantly in the 10 days after sepsis onset (A). In contrast, CRP and IL‐6 levels in preterm control neonates were low and within the range of normal adult values, but significantly increased in the initial days of sepsis (B and C). Bars represent medians and interquartile ranges. Significant differences were determined by Kruskal–Wallis test across all age groups and by *post hoc* Dunn's multiple comparison tests between subgroups (*p < 0.01, ***p < 0.0001). Dashed lines indicate mean values ± 2SD in healthy adults. (D) Receiver operating characteristic (ROC) curves for S100A8/A9, CRP, and IL‐6 demonstrate the superiority of CRP and IL‐6 over S100A8/A9 in sepsis detection at the onset of sepsis in neonates

Next, we investigated the influence of the following common sepsis risk factors on S100A8/A9 serum levels during the first 2 days of life in a cohort of 198 at birth healthy preterm infants (Table S2 and Methods in the Supporting Information): delivery by cesarean section (CS), birth weight (BW) of <1000 g, BW small for gestational age (SGA) (below the 10th percentile), male sex, insertion of a central venous line (CVL), and the need of invasive mechanical ventilation (IMV)^8^, ^9^ as detailed in the Supporting Information. Children born by CS had significantly lower S100A8/A9 levels compared to those born by vaginal delivery (VD) (Figure 2A). Among infants born by CS S100A8/A9 levels were higher after secondary CS compared to primary CS (Figure S1), suggesting dependence of perinatal S100A8/A9 levels on labor‐associated stress, which is in line with increased levels in trauma patients or marathon runners.^7^ In addition to the mode of delivery (MOD), also BW (Figure 2B), SGA status (Figure 2C), and sex (Figure 2D) were important determinants of the increase of S100A8/A9 serum levels at the beginning of life but not the need of CVL or IMV (Figure 2E and F). Subgroup analyses in preterm newborns delivered via CS, with a BW of <1000 g, SGA status, or male sex revealed that despite generally lower values (Figure 2A–D), S100A8/A9 levels at birth within these groups were still significantly lower in children with LOS compared to children that remained without LOS (Figure S2).

**FIGURE 2:**
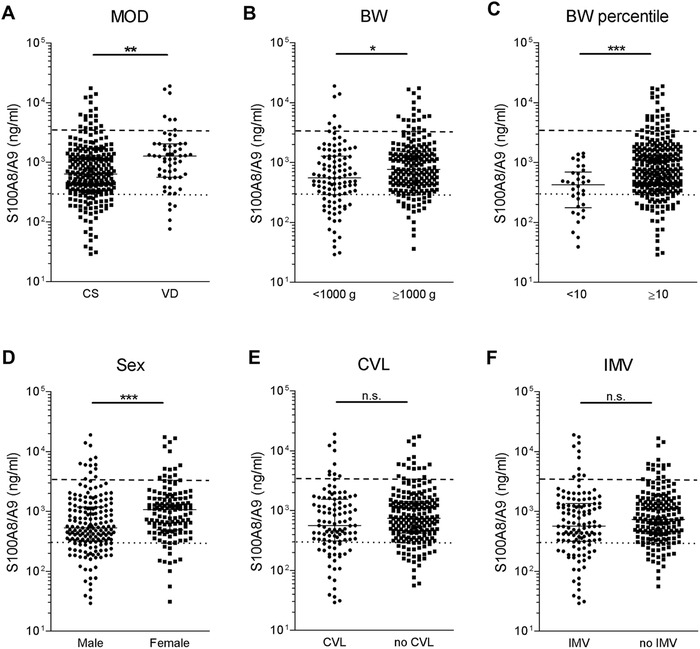
Association of serum S100A8/A9 in preterm newborn infants with variables related to birth and child conditions. (A–F) S100A8/A9 concentrations were determined in *n* = 314 serum samples obtained on the first two days of life from *n* = 198 preterm infants at 22–32 gestational weeks without signs of infection or inflammation. Levels were grouped according to (A) mode of delivery (MOD) (VD, vaginal delivery, *n* = 58; CS, cesarean section, *n* = 256), (B) birth weight (BW <1000 g *n* = 106; ≥1000 g *n* = 208), (C) BW percentile (<10th percentile *n* = 34; ≥10th percentile *n* = 280), (D) sex (male *n* = 179; female *n* = 135), (E) insertion of central venous line (CVL *n* = 107; no CVL *n* = 207), and (F) need of invasive mechanical ventilation (IMV *n* = 125; no IMV *n* = 189). Bars represent medians and interquartile ranges. *p < 0.01, **p< 0.001, ***p < 0.0001 (Mann–Whitney *U* tests). Horizontal lines indicate mean values ± 2SD in healthy term neonates (dashed) and healthy adults (dotted). Children born by CS had significantly lower S100A8/A9 levels compared to those born by vaginal delivery (VD) (A). Among infants born by CS S100A8/A9 levels were higher after secondary CS compared to primary CS (Figure S1). Furthermore, S100A8/A9 serum levels were significantly lower in extremely low BW infants with a BW of < 1000 g compared to infants with a BW ≥1000 g (B) and in small for gestational age (SGA) infants defined as a BW below the 10th percentile compared to infants appropriate for GA (Figure 2C). Also, the sex had an impact on initial S100A8/A9 serum levels, which were significantly lower in male than in female preterm newborns (D). In contrast, the presence of a CVL or need for IMV did not alter S100A8/A9 serum levels at the first two days after birth (E and F)

To test the value of serum S100A8/A9 at birth as independent sepsis risk marker, we then performed a nested model comparison while accounting for all identified confounding factors. This analysis validated that serum levels of S100A8/A9 at birth highly significantly and inversely correlated with the occurrence of sepsis, independent of the MOD, BW, SGA status, and sex (Figure 3A). This is in accordance with the sepsis‐protective function of S100A8/A9 in neonates shown previously.^6^, ^7^, ^10^ Calculating the effect sizes of all sepsis risk factors considered in this study and detailed in the Supporting Information revealed the strongest association of LOS with low S100A8/A9 levels compared to IMV, SGA status, low BW, male sex, MOD, and presence of CVL (Figure 3B). S100A8/A9 values within the normal range of adults, that is, below a cut‐off level of 300 ng/ml, increased the OR of LOS to 8.3‐fold (95%CI 4.15–16.72, *p *< 0.0001) with a positive predictive value of 0.92, a sensitivity of 0.89 and a specificity of 0.51. Above a S100A8/A9 level of 1000 ng/ml, the LOS risk decreased to 0.2 with a negative predictive value of 0.95 (OR 0.2, 95% CI 0.08–0.48, *p *= 0.0004). These cut‐off values might be valuable for neonatologists enabling them to identify newborn preterm infants at low risk of LOS and to avoid unnecessary empirical antibiotic treatment. In this regard, S100A8/A9 is superior to CRP and IL‐6 whose negative predictive power is limited due to the late peak of CRP after sepsis onset^2^ and the varying baseline levels of IL‐6 in neonates.^1^

**FIGURE 3:**
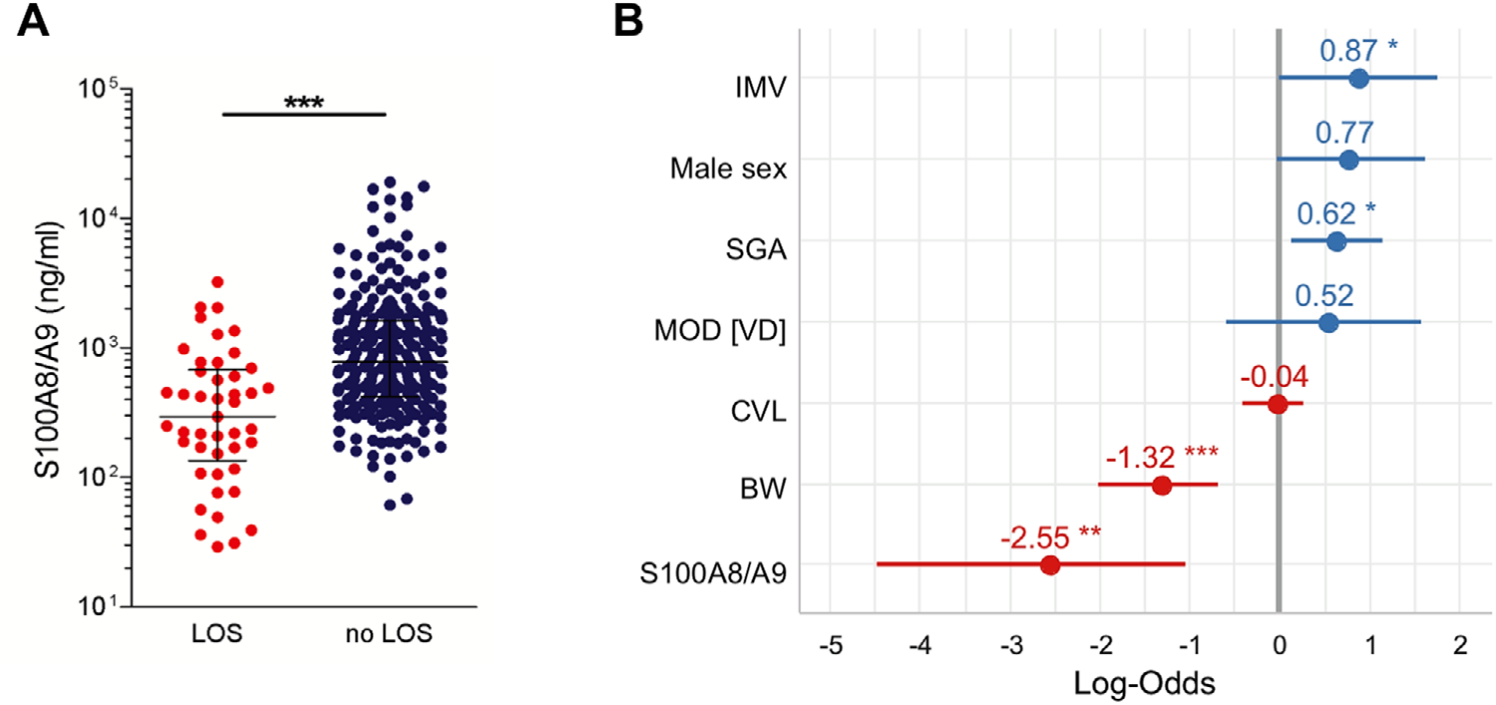
Low serum S100A8/A9 at birth predicts an increased risk of later development of sepsis in preterm newborn infants. (A and B) S100A8/A9 concentrations were determined in *n* = 314 serum samples obtained on the first two days of life from *n* = 198 preterm infants at 22–32 gestational weeks without signs of infection or inflammation. (A) Plotted are medians and interquartile ranges of S100A8/A9 levels grouped according to the occurrence of late‐onset sepsis (LOS *n* = 45; no LOS *n* = 269). The significance of the difference in S100A8/A9 levels between these groups was determined in a nested generalized linear mixed effect model to calculate the impact of S100A8/A9 on later LOS, when adjusting for mode of delivery (MOD), sex, birth weight (BW), BW percentile, central venous line (CVL), and invasive mechanical ventilation (IMV) (likelihood ratio test). ***p < 0.0005. (B) Effect sizes building a generalized linear model of indicated risk factors were plotted as log odds ratio. Male sex, MOD, and presence of CVL were not significantly related to an increased risk of LOS in our cohort. However, significant associations with LOS were found for IMV, SGA status, low BW, and low S100A8/A9 levels with the latter showing the strongest association with later LOS. *p < 0.05, **p < 0.01, ***p < 0.001

In summary, our data establish serum S100A8/A9 on the first 2 days of life as the first biomarker that reliably predicts the sepsis risk in preterm neonates, independent of patient characteristics. Useful S100A8/A9 cut‐off values are provided that stratify preterm infants into high or low sepsis risk groups at the earliest perceivable moment after birth. Analysing postnatal S100A8/A9 serum levels is a simple clinical assay that could ensure timely sepsis management reducing mortality rates and aid in avoiding unnecessary antibiotic treatment improving antibiotic stewardship in this vulnerable patient group.

This work was supported by the Interdisciplinary Centre of Clinical Research at the University of Münster [Vo2/011/19]; the Cluster of Excellence Cells in Motion and the Collaborative Research Centre 1009 [B08/B09]; and the Clinical Research Unit 342 [P3/P5] to TV and JR; by the Appenrodt Foundation; the Deutsche Forschungsgemeinschaft (DFG) [VI 538/6‐1]; and the DFG under Germany's Excellence Strategy—EXC 2155–390874280 [RESIST]; and the Volkswagen Foundation [Az 90005] to DV.

## AUTHOR CONTRIBUTIONS

Sabine Pirr, Louise Dauter, and Dorothee Viemann conceived and designed the study; Sabine Pirr, Louise Dauter, Bettina Bohnhorst, and Dorothee Viemann acquired the data and collected and prepared the samples for analysis; Sabine Pirr, Louise Dauter, Thomas Vogl, Thomas Ulas, Johannes Roth, and Dorothee Viemann performed the analyses and interpreted the data; Sabine Pirr, Louise Dauter, and Dorothee Viemann drafted the manuscript; and all authors critically revised the manuscript. Sabine Pirr and Louise Dauter share first authorship and contributed equally to this study and manuscript.

## CONFLICT OF INTEREST

The authors report no potential conflict of interest.

## DATA AVAILABILITY STATEMENT

De‐identified patient data from the results reported in this article will be made available for 5 years after publication upon request to the corresponding author. Researchers will need to provide a proposal and sign a data access agreement.

## Supporting information

Supporting Information
