# Supplementary material for: S100A8/A9 is the first predictive marker for neonatal sepsis
Source: Clin Transl Med. 2021 Apr 5;11(4):e338. doi: 10.1002/ctm2.338 (PMC8021540; doi:10.1002/ctm2.338)
Supplement: Supplementary file 1 — Supporting Information [file CTM2-11-e338-s001.docx]

**Supporting Information**

**Methods**

**Table S1.** Clinical characteristics of the matched case-control population of preterm infants

**Table S2.** Clinical characteristics of the preterm infant birth cohort

**Figure S1.** Labor-induced stress impacts on the height of S100A8/A9 serum levels in newborn preterm infants.

**Figure S2.** The association of low serum S100A8/A9 with later LOS remains unaffected from factors with decreasing impact on S100A8/A9 levels.

**METHODS**

**Definitions**

*Neonatal sepsis* was defined according to the criteria of the national infection surveillance system “NEO-KISS”^1^. *Clinical sepsis* was diagnosed in the presence of at least two clinical criteria (temperature >38°C or <36.5°C, tachycardia >200/min, occurrence or increase of hypoxemias, bradycardias or apneas, hemodynamic instability, hyperglycemia >10mmol/l, metabolic acidosis, greyish skin color, and prolonged reperfusion time) or one clinical and at least one laboratory sign (CRP >20mg/l, IL-6 >300ng/l, a ratio of immature to total neutrophils of >.2, white blood cell count <5/nl, and platelet count <100/nl) and antibiotic treatment for a minimum of five days, but no proof of causative agent in the blood culture. *Blood-culture proven sepsis* was defined as clinical sepsis with pathogen growth in the blood culture. If coagulase negative staphylococci (CoNS) were detected as single pathogen in the blood culture, two separate positive cultures were mandatory to consider CoNS as the sepsis causative agent^2^. *Early-onset sepsis (EOS)* was defined as sepsis occurring within the first 72 hours after birth, *late-onset sepsis (LOS)* was defined as sepsis after the first 72 hours of life.

*Gestational age (GA)* was calculated based on the last menstrual period. When early ultrasound at 11–13^+6^ week’s gestation using the fetal Crown-Rump-Length deviated more than seven days, dating was performed using ultrasound.

*Small for gestational age (SGA)* newborns were defined as having a birth weight (BW) below the 10^th^ percentile.

*Primary Cesarean section (CS)* was defined as section performed before onset of labor and *secondary CS* as section after the onset of labor according to uterine contractions recorded by tocography.

*Amnion infection syndrome* was defined as maternal fever (≥38°C) and at least two of the following clinical or laboratory signs: maternal leukocytosis (>15,000 cells/µl), maternal tachycardia (>100 bpm), fetal tachycardia (>160 bpm), uterine tenderness, or foul odor of amniotic fluid, while another maternal infection site has been excluded.

**Study population**

Clinical metadata and serum samples were prospectively collected from preterm infants with a GA of 22 to 32 weeks born at the Hannover Medical School from February 2012 to November 2018. Infants with major congenital malformation, inborn errors of metabolism, immunodeficiencies, or perinatal asphyxia, and births resulting from maternal trauma were excluded.

For the evaluation of S100A8/A9 as biomarker of neonatal sepsis, serum levels were determined in 75 blood samples obtained from 41 preterm infants at the onset of sepsis (day 0) when first clinical symptoms were evident and the following days (days one to ten) if blood was drawn for monitoring purposes. The mean age at diagnosis of sepsis was 3.0 ± 3.9 days. Thirty-one infants suffered from EOS and ten infants from LOS. Five cases (12.1%) were blood culture proven (one CoNS, two *Bacillus cereus*, one group B Streptococcus, one *Klebsiella aerogenes*). Control serum samples (n = 75) were obtained from routine or diagnostic peripheral blood drawings in infants without sepsis (n = 50) that were matched with the sepsis patients regarding GA, postnatal age, mode of delivery (MOD), sex, and BW (Table S1). In addition to S100A8/A9 levels, CRP concentrations were determined in all serum samples (n = 150) and IL-6 concentrations were measured in 68 samples of the sepsis group and 66 samples of the control group.

To analyze the association between S100A8/A9 serum levels at the beginning of life and the later occurrence of LOS, 314 serum samples from 198 preterm infants were collected in a preterm infant birth cohort from cord blood (day 0) and peripheral blood drawings at day one or two of life. Infants born with amnion infection syndrome or suffering from EOS have been excluded from this analysis. For the collection of clinical metadata children were followed until discharge. Clinical characteristics of the preterm study cohort are summarized in Table S2. LOS occurred in 25 infants (12.6%) whereof eight cases (32.0%) were blood culture proven (one *Staphylococcus aureus*, one CoNS, two *Bacillus cereus*, one *Escherichia coli*, two *Klebsiella pneumoniae*, two *Klebsiella aerogenes*).

**Measurement of S100A8/A9, CRP and IL-6 concentrations**

Serum samples were centrifuged at 3000 × g at room temperature for ten minutes and either stored at -80°C for later S100A8/A9 analysis or used immediately for the analysis of CRP and IL-6. S100A8/A9 was determined by an in-house ELISA as described previously^3^. CRP concentrations were determined by particle-enhanced turbidimetric immunoassay (PETIA) with Cobas 8000, module c701 (Hoffmann-La Roche, Basel, Switzerland). IL-6 concentrations were measured via ElectroChemiLuminescence immunoassay (ECLIA) with Cobas 8000, module e801 (Hoffmann-La Roche, Basel, Switzerland).

**Statistical analysis**

For subgroup comparisons of levels of S100A8/A9, CRP and IL-6 Mann-Whitney *U* tests, Kruskal-Wallis tests with *post-hoc* Dunn’s multiple comparison test and unpaired *t*-test with Welch’s correction for unequal variances were applied as indicated in the figure legends. For the comparison of S100A8/A9, CRP and IL-6 as biomarker at the onset of neonatal sepsis receiver operating characteristic (ROC) curves were build. Statistical analyses were carried out using GraphPad Prism software (version 5; GraphPad software, San Diego, CA, USA). The association between S100A8/A9 serum levels on day 0 to 2 and the occurrence of LOS was assessed by employing a nested model test building generalized linear mixed effect models (R lme4 package) of measured data using a Gamma distribution error model and setting zero values to a pseudocount floor of 1-e-10. The model with S100A8/A9, MOD, sex, BW, BW percentile, central venous line (CVL), and invasive mechanical ventilation (IMV) was contrasted to a model containing MOD, sex, BW, BW percentile, CVL, and IMV only to test for main independent effect of S100A8/A9 using a likelihood ratio test. The effect sizes of S100A8/A9, MOD, sex, BW, BW percentile, CVL, and IMV on the occurrence of LOS were calculated by employing a generalized linear model and visualized using the R package sjplot (v2.8.3; URL:https://CRAN.R-project.org/package=sjPlot) and function plot-model^4^ using default parameters. Odds ratios (OR), their 95% confidence intervals (CI) and significances were calculated by Chi^2^-test. A *P* value <.05 was considered statistically significant.

**Ethics statement**

Protocols were approved by the Institutional Review Board of the Hannover Medical School (no. 6031-2011, no. 6031-2015, Research Obstetrics Biobank no. 1303-2012) and were in accordance with the Helsinki Declaration (1964, amended most recently in 2008) of the World Medical Association. Written informed consent was obtained from the parents of participating infants.

**Table S1.** Clinical characteristics of the matched case-control population of preterm infants

| **Parameter** | **Controls**  **(n = 50)** | **Sepsis^a,b^**  **(n = 41)** | ***P* value**^c^ |
| --- | --- | --- | --- |
| Gestational age in weeks, mean (±SD) | 28.0 (2.4) | 27.9 (2.5) | .9079 |
| Postnatal age in days, mean (±SD) | 3.7 (4.1) | 3.8 (4.1) | .7300 |
| Birth weight in gram, mean (±SD) | 1023 (347) | 1068 (423) | .9428 |
| SGA, No. (%) | 9 (18) | 8 (20) | .8593 |
| Female Sex, No. (%) | 20 (40) | 16 (39) | .9287 |
| Cesarean section, No. (%) | 42 (84) | 34 (83) | .8963 |
| 5 min Apgar score, mean (±SD) | 7.8 (1.1) | 7.5 (.9) | .0867 |
| 10 min Apgar score, mean (±SD) | 8.7 (.8) | 8.5 (.9) | .2369 |
| Umbilical artery pH, mean (±SD) | 7.3 (.1) | 7.3 (.1) | .8076 |

^a^ Mean day of life at diagnosis 3.0 ± 3.9.

^b^ Clinical sepsis n = 36, blood culture proven sepsis n = 5 (12.2%; 1 group B Streptococcus, 1 CoNS, 2 *Bacillus cereus*, 1 *Klebsiella aerogenes*).

^c^ Mann-Whitney *U* test.

**Table S2.** Clinical characteristics of the preterm infant birth cohort

| **Parameter** | **Controls**  **(n = 173)** | **LOS^a,b^**  **(n = 25)** | ***P* value**^c^ |
| --- | --- | --- | --- |
| Gestational age in weeks, mean (±SD) | 28.9 (2.2) | 26.9 (2.1) | .0001 |
| Birth weight in gram, mean (±SD) | 1196 (380) | 904 (311) | .0003 |
| Female sex, No. (%) | 72 (42) | 6 (24) | .0932 |
| Cesarean section, No. (%) | 142 (82) | 21 (84) | .8168 |
| CVL, No. (%) | 53 (31) | 16 (64) | .0011 |
| IMV, No. (%) | 62 (36) | 19 (76) | .0001 |
| 5 min Apgar score, mean (±SD) | 7.9 (1.2) | 7.4 (1.2) | .0629 |
| 10 min Apgar score, mean (±SD) | 8.7 (.8) | 8.5 (.8) | .1085 |
| Umbilical artery pH^,^ mean (±SD) | 7.32 (.08) | 7.35 (.07) | .0605 |

^a^ Mean day of life at diagnosis 14.0 ± 9.9.

^b^ Clinical sepsis n = 17, blood culture proven sepsis n = 8 (32.0%; 1 *Staphylococcus aureus*, 1 CoNS, 2 *Bacillus cereus*, 1 *Escherichia coli*, 2 *Klebsiella pneumoniae*, 1 *Klebsiella aerogenes*).

^c^ Mann-Whitney *U* test.

**Figure S1 (related to Figure 2).** Labor-induced stress impacts on the extent of S100A8/A9 serum levels in newborn preterm infants.


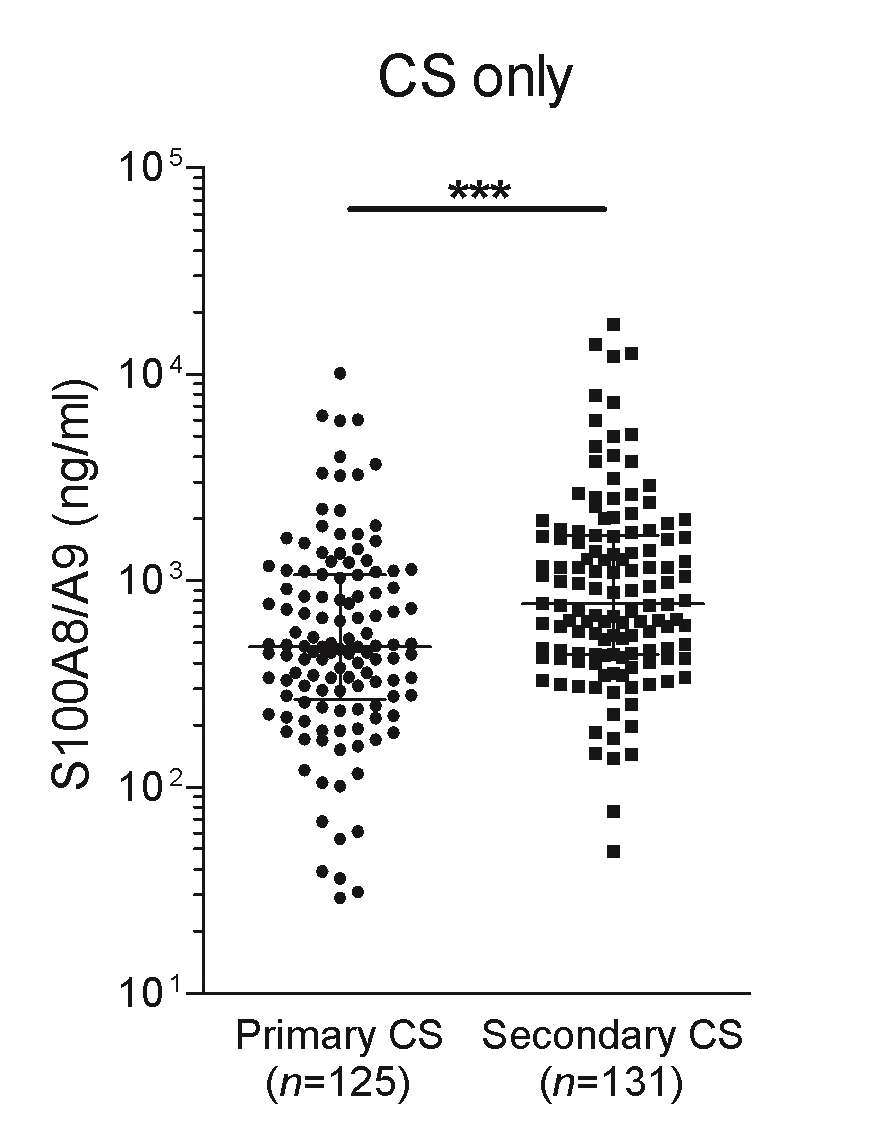


S100A8/A9 concentrations were determined in n = 256 serum samples obtained on the first two days of life from n = 163 preterm infants at 22 to 32 gestational weeks born by cesarean section (CS) without signs of infection or inflammation. Plotted are medians and interquartile ranges of S100A8/A9 levels grouped according to the absence (primary CS, n = 125) and presence (secondary CS, n = 131) of labor during delivery. ****P* <.0001 (Mann-Whitney *U* test).


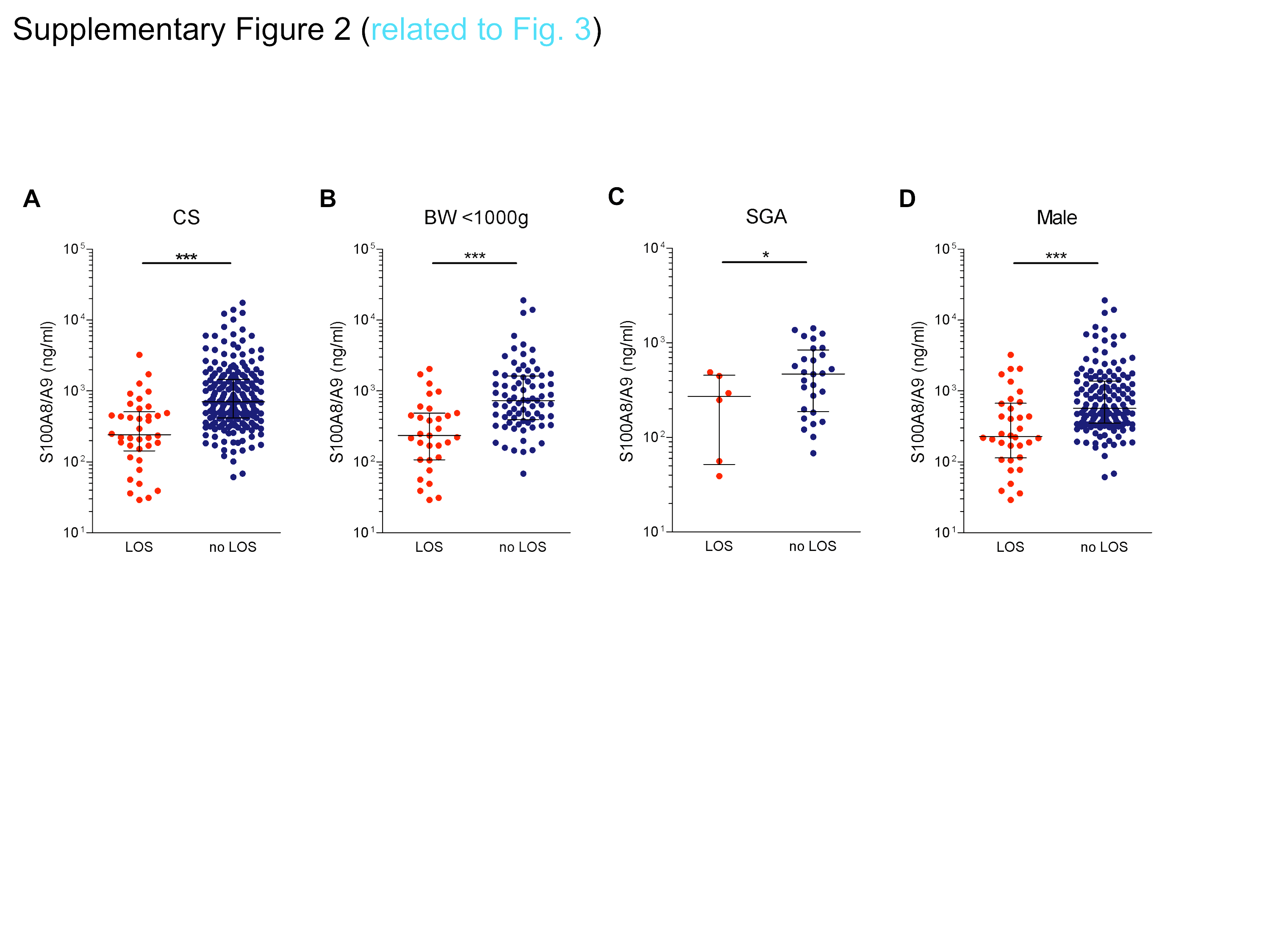
**Figure S2 (related to Figure 3).** The association of low serum S100A8/A9 with the development of LOS remains unaffected from factors with decreasing impact on S100A8/A9 levels.

**A–D**, S100A8/A9 concentrations were determined in n = 314 serum samples obtained on the first two days of life from n = 198 preterm infants at 22 to 32 gestational weeks without signs of infection or inflammation. Plotted are median and interquartile range of S100A8/A9 levels grouped according to the occurrence of LOS in newborns **A**, born by CS (LOS n = 38; no LOS n = 218), **B**, with extremely low birth weight (BW <1000 g) (LOS n = 31; no LOS n = 78), **C**, small for gestational age (SGA) status (LOS n = 6; no LOS n = 28), and **D**, of male sex (LOS n = 34; no LOS n = 145). **P* <.05, ****P* <.0001 (Mann-Whitney *U* tests (**A, B, D**) and unpaired *t*-test with Welch’s correction (**C**)).

**References**

1. Leistner R, Piening B, Gastmeier P, Geffers C, Schwab F. Nosocomial infections in very low birthweight infants in Germany: current data from the National Surveillance System NEO-KISS. *Klin Padiatr.* 2013;225:75–80. doi: 10.1055/s-0033-1334886.
2. Healy CM, Baker CJ, Palazzi DL, Campbell JR, Edwards MS. Distinguishing true coagulase-negative Staphylococcus infections from contaminants in the neonatal intensive care unit. *J Perinatol.* 2013;33:52e8. doi: 10.1038/jp.2012.36.
3. Vogl T, Tenbrock K, Ludwig S, et al. Mrp8 and Mrp14 are endogenous activators of Toll-like receptor 4, promoting lethal, endotoxin-induced shock. *Nat Med.* 2007;13:1042–9. doi: 10.1038/nm1638.
4. Lüdecke D. sjPlot: Data Visualization for Statistics in Social Science. (2020) Version 2.8.4. URL:https://doi.org/10.5281/zenodo.1308157. Accessed November 27, 2020. doi: 10.5281/zenodo.2400856.
